# Within-subject alterations in CSF and blood flow dynamics following rhythm-control intervention in atrial fibrillation: An exploratory multimodal MRI study

**DOI:** 10.1016/j.nicl.2026.104029

**Published:** 2026-07-03

**Authors:** Sabine Hofer, Marlena Schnieder, Leonie Polster, Angelika S. Bader, Vitali Telezki, Peter Dechent, Mathias Bähr

**Affiliations:** a Department of Neurology, University Medical Center Göttingen, Germany; b Department of Geriatrics, University Medical Center Göttingen, Germany; c Department of Cardiovascular Imaging, Center for Clinical Radiology Imaging, University Medical Center, Göttingen, Göttingen, Germany; d Cluster of Excellence Multiscale Bioimaging: from Molecular Machines to Networks of Excitable Cells (MBExC2067), University of Göttingen, Germany; e German Center for Cardiovascular Research (DZHK), Partner Site Lower Saxony, Göttingen, Germany; f MR-Research in Neurosciences, Department of Cognitive Neurology, University Medical Center Göttingen, Germany

**Keywords:** Brain, Neurofluid dynamics, CSF oscillation, Atrial fibrillation, Rhythm control, MRI

## Abstract

**Background:**

Cardiac- and respiration-driven vascular pulsations influence cerebrospinal fluid (CSF) oscillations and neurofluid dynamics and are considered relevant for perivascular fluid transport and brain homeostasis. In atrial fibrillation (AF), irregular cardiac rhythm alters cerebral hemodynamics and may affect CSF flow dynamics. This exploratory pre–post pilot study investigated physiological changes in CSF and blood flow dynamics following AF intervention.

**Methods:**

This pilot study included 7 patients with AF, using a 3 T MRI system. To capture CSF and blood flow, real-time phase-contrast flow MRI was employed in the aqueduct, the internal carotid artery, the jugular vein and the sagittal sinus. T1-weighted MRI and fast T1 mapping characterized the tissue anatomy and integrity and EPI diffusion assessed fluid motion along the perivascular space. Artefact-free STEAM diffusion described whole-brain CSF dynamics. Physiological data were recorded simultaneously during MRI.

**Results:**

Before intervention, AF patients exhibited reduced CSF flow and irregular CSF oscillatory patterns. After intervention, CSF dynamics shifted toward more periodic, cardiac-dominated oscillatory patterns, with increased CSF flow volume, consistent with altered neurofluid dynamics following restoration of sinus rhythm. Blood flow in the internal carotid artery, jugular vein, and sagittal sinus similarly shifted toward sinus rhythmic patterns, while DTI-ALPS, quantitative T1 measures, brain volume, ventricular size, and white matter integrity remained unchanged.

**Conclusion:**

Within-subject comparisons indicated that restoration of sinus rhythm was accompanied by measurable alterations in CSF and blood flow dynamics.

## Highlights

• Multimodal MRI assessed CSF and blood flow dynamics in AF.
• Sinus rhythm restoration altered CSF and blood flow patterns.
• CSF oscillations became more cardiac-frequency dominated after treatment.
• Oscillatory CSF flow increased after restoration of sinus rhythm.

## Graphical abstract

**Figure:**
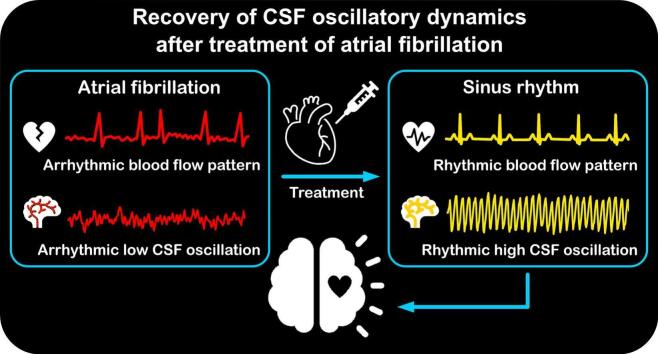

## 1 Introduction

Waste clearance, dynamic fluid exchange and perivascular fluid transport are essential processes for maintaining brain homeostasis. The description of the glymphatic system (GS) has significantly advanced our understanding of cerebral waste-clearance and brain homeostasis (Hablitz and Nedergaard, 2021). In this model, astrocytic endfeet enriched with aquaporin-4 (AQP4) water channels enable cerebrospinal fluid (CSF) to enter the brain parenchyma, mix with interstitial fluid, and subsequently exit along perivenous spaces and meningeal and cervical lymphatic vessels (Nedergaard, 2013). The exchange of solutes between CSF and interstitial fluid is primarily driven by arteriolar pulsations, vasomotion, and respiratory activity (Rasmussen et al., 2022). Cardiac-related vascular pulsations generate oscillatory CSF motion that is considered an important contributor to perivascular fluid transport. Respiratory activity further regulates these oscillations by modulating venous outflow and pressure gradients, thereby influencing fluid movement through perivascular pathways (Alperin and Burman, 2024; Burman and Alperin, 2024; Mestre et al., 2018; Dreha-Kulaczewski et al., 2015; Kollmeier et al., 2022a).

While the glymphatic concept provides an important conceptual model, the precise mechanisms of neurofluid dynamics in humans remain incompletely understood and are subject to ongoing debate. Cerebral blood flow and vascular pulsatility are key determinants of brain physiology and have been consistently linked to cognitive function, with reduced perfusion associated with cognitive decline across aging and cerebrovascular disease populations (Leeuwis et al., 2018; van Dinther et al., 2024). In parallel, alterations in CSF flow dynamics have been associated with impaired cognition, suggesting that neurofluid dynamics may contribute to brain function (Attier-Zmudka et al., 2019).

When the heartbeat becomes irregular, such as in atrial fibrillation (AF), the wave dynamics of the vascular walls are disrupted, interfering with the normal rhythm of arteriolar systolic pulsations. This arrhythmia leads to altered cerebral hemodynamics and vascular pulsatility, which may influence both cerebral perfusion and CSF flow dynamics. AF affects millions of people worldwide and is associated with an increased risk of stroke, heart failure, and death (Chugh et al., 2014). AF is characterized by irregular, usually fast, atrial activity with consequent deterioration of atrial function. AF is classified as paroxysmal, persistent, or permanent, with the latter associated with worse prognosis. The primary aims of treatment for AF are stroke prevention (i.e., oral anticoagulation), symptom management with heart rate or rhythm control, and management of cardiovascular comorbidities. Apart from stroke, AF has been increasingly associated with cognitive decline (Madhavan et al., 2018). Asymptomatic and symptomatic macro- and microcerebral embolism have been proposed as potential causes for AF-associated cognitive impairment (Blum and Conen, 2023a), but this remains an unproven hypothesis. Moreover, it has been suggested that the irregular and aperiodic blood flow associated with AF could lead to cerebral microvascular damage. In addition to embolic mechanisms, AF-related alterations in cerebral perfusion and vascular dynamics may represent an important pathway contributing to cognitive decline. AF-related alterations in vascular pulsatility and cerebral blood flow may also influence CSF oscillatory patterns and neurofluid dynamics. The extent to which these physiological alterations translate into changes in perivascular fluid transport in humans remains unclear and warrants further investigation.

A recent study reported that the DTI-ALPS index (diffusion tensor image analysis along the perivascular space; (Taoka et al., 2017)), used as a surrogate marker of perivascular diffusivity, showed lower values in patients with AF and appeared to partially mediate the statistical association between AF and cognitive decline (Guo et al., 2025). However, the DTI-ALPS index is increasingly recognized as a non-specific marker that may reflect tissue microstructure composition rather than exclusively perivascular fluid transport (Ringstad, 2024), and this measure should therefore be interpreted with caution.

MRI technology is a valuable tool for investigating neurofluid dynamics in humans in vivo (Naganawa et al., 2024; Taoka and Naganawa, 2020). To obtain a comprehensive overview of neurofluid physiology, it is necessary to employ different techniques covering multiple aspects of cerebrovascular flow and CSF dynamics (Kamagata et al., 2023).

In this context, fast real-time phase-contrast MRI enables direct measurement of cardiac- and lower frequency-driven CSF and blood flow oscillations with high temporal resolution, allowing assessment of flow volumes, velocity, and directionality across key vascular and CSF regions (Burman and Alperin, 2024; Dreha-Kulaczewski et al., 2015; Kollmeier et al., 2022a; Maier et al., 2018). When combined with quantitative structural imaging (Merrem et al., 2017; Voit et al., 2021; Wang et al., 2015) that assess tissue integrity and fluid dynamics in the whole brain, as well as physiological recordings that monitor cardiac and respiratory inputs (Bader et al., 2025), this multimodal approach enables integrated assessment of vascular and CSF oscillatory dynamics in vivo (Hofer et al., 2025). Together, these complementary measurements provide a physiological characterization of neurofluid dynamics and associated processes potentially relevant to perivascular fluid transport.

Accordingly, this study addressed two key questions: (i) whether blood-flow and CSF-flow dynamics altered during atrial fibrillation change following individualized rhythm-control treatment, and (ii) whether oscillatory CSF volume changes after restoration of sinus rhythm.

Given the exploratory nature this study was designed to characterize physiological alterations within individuals rather than to establish clinical efficacy or long-term neurological consequences.

## 2 Material and methods

The study was approved by the ethics committee of the University Medical Center Göttingen (# 37/3/19) and written informed consent was obtained from each subject. The study was in compliance with the Declaration of Helsinki.

### 2.1 Subjects

Seven patients with AF participated in this MRI study. Patients were recruited from the Department of Cardiology and underwent MRI examinations before and after clinically indicated rhythm-control treatment, including electrical cardioversion or pulmonary vein ablation/isolation procedures. Electrical cardioversion restores sinus rhythm acutely via synchronized direct-current shock, whereas pulmonary vein ablation targets arrhythmogenic foci to achieve more durable rhythm control. Despite their different mechanisms and time courses, both interventions aim to re-establish organized atrial contraction and improve cardiac output. The study did not influence treatment allocation, which was determined independently based on clinical indication. AF was confirmed by electrocardiogram (ECG) prior to inclusion, and restoration of sinus rhythm was verified by ECG after intervention. Participants were excluded if they had contraindications to MRI, including implantation of non–MRI-compatible cardiac devices (e.g., pacemaker or implantable cardioverter-defibrillator). Subjects were also excluded in cases of coronary artery disease with significant wall motion abnormalities and heart failure with an ejection fraction <40%. Furthermore, participants with ischemic or hemorrhagic stroke were excluded as well as patients with neurological disorders potentially affecting brain structure, cognition, or cerebrospinal fluid dynamics, including neurodegenerative diseases, demyelinating disorders, or other central nervous system pathologies. If any of the exclusion criteria occurred between the two measurements, participants were excluded from the study. These criteria were applied to minimize potential confounding factors affecting neurovascular physiology and CSF dynamics. Baseline clinical characteristics, cardiovascular risk factors, and medication were recorded at study inclusion.

All participants accomplished a telephone-Montreal cognitive assessment (T-MoCA) to assess cognition (Katz et al., 2021; Zietemann et al., 2017). The conventional MoCA is a screening tool for mild cognitive impairment, administered in person and scored on a 30-point scale. In contrast, the telephone-based (t-MoCA) omits visually dependent and drawing-based components (e.g., visuospatial/executive tasks) due to remote administration constraints, resulting in a lower maximum achievable score (22 points). Because of these structural differences, normative thresholds for “cognitive normality” are substantially lower for t-MoCA than for in-person MoCA assessments; healthy performance on the t-MoCA typically corresponds to scores in the upper teens to low twenties, rather than >26 as in the conventional MoCA.

### 2.2 Magnetic resonance imaging

All data were acquired on a 3 Tesla MR system (Magnetom Prisma Fit, Siemens Healthineers, Erlangen, Germany) using a 64-channel head coil. To analyze different aspects of the GS function, we used an MRI protocol similar to acquisition techniques published previously (Hofer et al., 2025). Additionally, an electrocardiogram (ECG) and pulse (PPG) monitoring system, as well as a breathing belt integrated into the MRI system, were used to assess the physiological state of the subjects throughout the entire MR session.

Real-time phase-contrast flow MRI was implemented using highly undersampled radial FLASH acquisitions combined with a timing-optimized gradient scheme (Joseph et al., 2012; Untenberger et al., 2015). Velocity encoding was performed using alternating flow-compensated and flow-encoded acquisitions. Quantitative velocity maps were obtained from the corresponding phase differences during model-based reconstruction, as previously described for highly undersampled radial real-time phase-contrast MRI (Bernstein et al., 1998; Kollmeier et al., 2022b; Tan et al., 2017). Within this framework, image content and coil sensitivities are jointly estimated, while temporal regularization exploits continuity between successive frames, enabling stable reconstruction from a low number of radial spokes with high spatiotemporal fidelity (Frahm et al., 2019). No dual-VENC or multi-VENC approaches were applied.

For CSF measurements, a field of view (FOV) of 192 mm was applied, whereas blood flow imaging of the internal carotid artery (ICA), the jugular vein (JV) and the sagittal sinus were performed with an FOV of 160 mm. Corresponding image matrices were 256 and 200, yielding in-plane spatial resolutions of 0.75 × 0.75 mm^2^ and 0.8 × 0.8 mm^2^, respectively. CSF flow acquisitions were conducted with a slice thickness of 5 mm, flip angle of 12°, repetition time (TR) of 5.68 ms, and echo time (TE) of 4.61 ms. For blood flow measurements, the slice thickness was increased to 6 mm, while TR and TE were reduced to 4 ms and 2.79 ms, respectively, with the flip angle kept constant. Velocity encoding strength (VENC) was adjusted according to the peak velocities of CSF and blood. Low VENC settings of 10 cm s^−1^ were used for all CSF measurements, providing high sensitivity without evidence of phase wrapping in the present dataset. Blood flow measurements were required with a higher VENC of 100 cm s^−1^. 11 radial spokes per frame were used for CSF flow and 5 spokes for blood flow, resulting in temporal resolutions of 125 ms (8 frames s^−1^) for aqueductal CSF flow and 40 ms (25 frames s^−1^) for blood flow in the internal carotid artery, jugular vein, and sagittal sinus. Real-time visualization of the phase-contrast images allowed immediate quality control during acquisition. Each measurement comprised 500 magnitude images and 500 corresponding velocity maps for CSF flow, and 1500 magnitude images and velocity maps for blood flow, acquired during one minute of normal free breathing without cardiac gating. The protocol was consistent with previous studies (Dreha-Kulaczewski et al., 2017; Hofer et al., 2025; Kollmeier et al., 2022a; Maier et al., 2018; Vikner et al., 2024).

A VENC of 100 cm s^−1^ was used for blood-flow measurements based on prior validation and to avoid phase wrapping in the presence of potentially high peak velocities. While prior work has employed lower VENC settings for the sagittal sinus (e.g., 40 cm s^−1^) compared to the internal jugular vein (70 cm s^−1^) to optimize velocity-to-noise ratio in each compartment (Laganà et al., 2022), a uniform VENC was applied here to prioritize longitudinal consistency and avoid phase wrapping under potentially variable AF-related flow conditions. The trade-off is reduced velocity-to-noise ratio in lower-velocity venous compartments such as the sagittal sinus, which may have affected flow quantification accuracy in that vessel.

Diffusion-weighted MRI was performed using a single-shot stimulated echo acquisition mode (STEAM) sequence with strong radial undersampling (Merrem et al., 2017; Voit et al., 2021). Data were acquired along 6 diffusion-encoding directions with a total scan duration of 2 min 30 s. The measurement parameters were as follows: FOV 224 mm, resolution 1 × 1 × 3 mm^3^, slices 57, spokes 17, TR 7.52 ms, and TE 4.54 ms, b = 0, 500, 1000 s mm^−2^. The respective mean DWI and ADC maps were reconstructed via the real-time pipeline automatically (Voit et al., 2021).

Quantitative T1 mapping was performed using a single-slice T1FLASH acquisition covering the region used for DTI-ALPS index calculation and adjacent white matter. Images were acquired with an in-plane resolution of 0.75 × 0.75 mm^2^, a slice thickness of 3 mm, and a FOV of 224 mm (TR 3.51 ms, TE 2.24 ms, flip angle 6°) (Wang et al., 2016, 2015). Radial sampling employed a small golden-angle trajectory (20.89°). The acquisition of 62 images then resulted in a measuring time of 4 s per T1 map (Roeloffs et al., 2016). T1 relaxation maps were reconstructed automatically immediately after acquisition and displayed directly on the MRI system.

Vascular anatomy was visualized using a fast-angiographic protocol visualizing the ICA and JV. A strongly T1-weighted volumetric acquisition of the neck was performed with 1 mm^2^ in-plane resolution. The method is derived from a motion-robust real-time volume coverage approach and enables visualization of the arterial or venous anatomy within approximately 30 s. This scan was used for rapid vessel localization and accurate slice prescription (Voit et al., 2020) for further real-time phase-contrast measurements. Volume coverage was achieved using a highly undersampled radial gradient-echo sequence with rapid cross-sectional acquisition. For ICA imaging, a field of view of 192 mm was used with 326 measurements, a slice thickness of 4 mm, TR of 3.11 ms, TE of 1.97 ms, and a flip angle of 30°. Imaging of the JV employed a reduced slice thickness of 2 mm, with TR/TE of 3.83/2.47 ms and 395 measurements to ensure adequate coverage of the venous lumen. Maximum intensity projections enabled visualization of the vessel anatomy. To preferentially highlight arterial inflow or venous outflow, volume acquisition was performed in a cranio-caudal direction for arterial imaging and reversed for venous imaging.

Whole brain T1-weighted datasets were acquired with 1 mm^3^ isotropic resolution (MPRAGE, TR 2250 ms, inversion time (TI) 900 ms, TE 3.3 ms, flip angle 9°, FOV 256 mm, 176 sagittal sections).

Slice positioning for aqueductal CSF flow and sagittal sinus blood flow measurements were acquired on T1-weighted anatomical images. The aqueduct was identified on mid-sagittal slices, and the imaging plane was positioned perpendicular to its course. Similarly, the sagittal sinus was localized on sagittal images, and slice orientation was adjusted orthogonally to the vessel trajectory to ensure accurate flow quantification. Slice positioning for flow measurements was performed individually based on vessel anatomy rather than fixed external landmarks. Imaging planes were placed above the carotid bifurcation and aligned orthogonally to straight vessel segments to ensure accurate phase-contrast flow quantification. Because ICA and JV do not consistently follow parallel pathways, separate acquisitions with individually adjusted slice orientations were performed for arterial and venous measurements. For longitudinal consistency, slice positioning was manually reproduced using the same anatomical landmarks and orientation criteria in pre- and post-treatment scans.

Additional diffusion-weighted imaging was performed using a single-shot echo-planar imaging (EPI) sequence. Whole-brain coverage was achieved with 65 slices at an isotropic spatial resolution of 2 mm^3^. Diffusion sensitization was applied along 64 diffusion directions with a b-value of 1000 s mm^−2^, complemented by a single non–diffusion-weighted reference image (b = 0 s mm^−2^). To enable correction of susceptibility-related geometric distortions, an additional acquisition with reversed phase-encoding polarity was included (Andersson et al., 2003; Smith et al., 2004).

All data were stored and managed within our research data management system tool (Fitschen et al., 2019; Telezki et al., 2024).

### 2.3 MRI data analysis

**Fig. 1:**
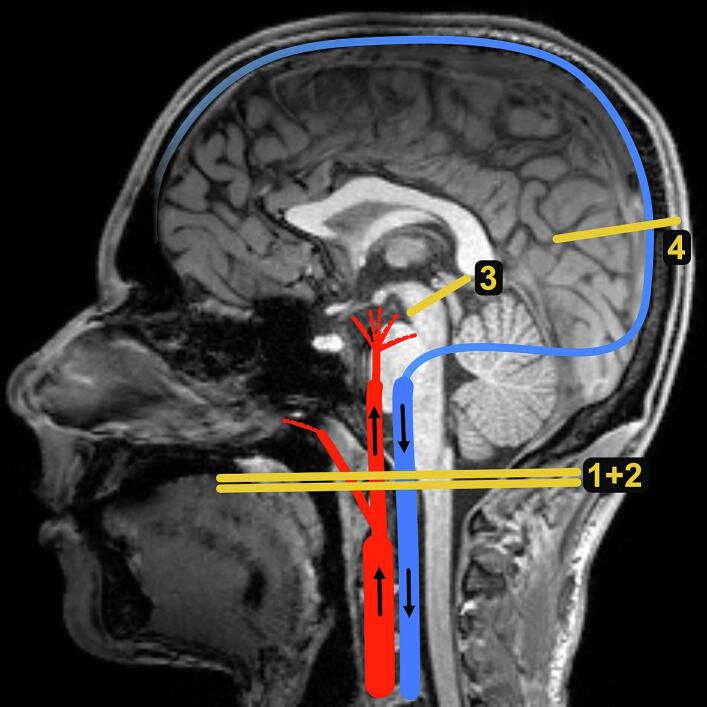
Schema of slice positions for the respective phase-contrast flow measurements orthogonal to the flow direction, overlaid on a T1 weighted sagittal image. Yellow lines indicate the respective slices positions of the ICA and JV above the bifurcation (1 + 2), the aqueduct (3) and the sagittal sinus (4). Red color: arteries; blue color: veins. Arrows indicate the flow direction in the arteries and veins.

Fig. 1 shows a T1-weighted sagittal anatomical image illustrating the positions of the measurement slices used for the phase-contrast acquisitions. Regions of interest (ROIs) were subsequently defined on the phase-contrast images in the ICA, JV, cerebral aqueduct, and sagittal sinus. Although scans were not spatially registered to avoid interpolation-related bias, ROI positioning followed predefined clearly visible anatomical landmarks, e.g., the aqueduct, ICA, JV, and the sagittal sinus. This approach prioritizes quantitative signal integrity over voxel-wise spatial correspondence.

Real-time flow MRI datasets were quantitatively analyzed using CaFuR software (Fraunhofer Mevis, Bremen, Germany) (Chitiboi et al., 2014). Following manual initialization of a single ROI, automatic segmentation of flow signals was performed on the real-time image series as described previously (Kollmeier et al., 2022a; Maier et al., 2018). This approach enables dynamic tracking of the vessel position over time and compensates for in-plane motion, ensuring consistent ROI placement throughout the measurement. ROIs covering the ICA were analyzed bilaterally for the blood vessels. Jugular venous flow was analyzed bilaterally whenever both veins could be reliably identified and quantified. However, substantial inter-individual anatomical asymmetry was observed across subjects. In several cases, one JV represented the clearly dominant drainage pathway, while the contralateral vein was markedly hypoplastic, poorly visible, or not consistently identifiable within the measurement plane. Under these conditions, reliable contouring and reproducible flow quantification of the smaller vessel were not feasible. Therefore, analysis was restricted to the clearly identifiable dominant JV in these subjects. While this approach may limit direct comparability of venous flow volumes across subjects, it was considered the most robust and reproducible strategy for within-subject flow analysis in the presence of pronounced venous asymmetry.

Heart rate was derived from the pulsatile ICA flow signal obtained by real-time phase-contrast MRI. Peak detection and estimation of mean pulsation rates were performed using the automated analysis tools implemented in the CaFuR software. Mean pulsation rates were derived from temporal peak-to-peak intervals averaged over the entire acquisition period.

To account for the bidirectional and oscillatory nature of aqueductal CSF flow, we quantified the cumulative flow by integrating the flow over time. This metric represents the oscillatory CSF volume (ml), i.e., the cumulative CSF flow volume in both cranial and caudal directions during the acquisition. The resulting values were normalized to the acquisition duration and expressed in ml min^−1^. In the present study, acquisition times were approximately 60 s. This measure does not represent CSF production or net CSF flow, but rather the magnitude of bidirectional aqueductal CSF oscillations.

The relative contributions of low frequency and respiration components and cardiac components to flow signals were quantified using a power ratio analysis (Yildiz et al., 2017). The respiratory component (r) was defined as spectral power within the frequency range of 0–0.5 Hz, representing a low-frequency band dominated by respiratory contributions, following previous work (Yildiz et al., 2017) and consistent with our prior study (Hofer et al., 2025). Prior to spectral analysis, the temporal mean of the flow signal was removed to eliminate the constant flow component at 0 Hz. This range captures the dominant non-cardiac components of the flow signal but may additionally include very-low-frequency fluctuations (e.g., vasomotor activity), particularly below 0.1 Hz. The cardiac component (c) was defined from the integrated spectral power within a frequency window centered around the dominant spectral peak identified between 0.75 and 2 Hz. After automatic detection of the maximum spectral amplitude within this range, cardiac power was calculated using a bandwidth of ±0.25 Hz around the detected peak. A symmetric bandwidth of ±0.25 Hz was selected to provide robust integration of cardiac-related spectral power despite minor frequency variability and spectral broadening, particularly under irregular rhythm conditions such as atrial fibrillation. Consequently, closely adjacent spectral peaks jointly contributed to the cardiac component within the same integration window. Visual inspection was performed only as a quality-control step to confirm adequate capture of the dominant cardiac-related spectral contribution without manual adjustment of the bandwidth. Visual quality control was performed by a single rater (S.H.) blinded to treatment status.

Lower r/c values reflect a higher relative contribution of cardiac-frequency components within the flow signal rather than a direct measure of oscillation amplitude. In the present dataset, lower r/c values were associated with more regular and visually more pronounced cardiac-driven oscillatory flow patterns following restoration of sinus rhythm. The r/c index was interpreted as a surrogate marker of the relative waveform composition, whereas the quantified oscillatory CSF volume provided complementary information regarding the magnitude of bidirectional CSF movement.

The r/c index for the blood vessels was evaluated in vessels with clear visualization and flow direction oriented orthogonal to the imaging slice (ICA r/c index; JV r/c index). Ratios greater than one indicate predominance of respiratory modulation, whereas values below one reflected primarily cardiac-driven flow.

Although CSF flow was sampled at a lower temporal resolution (8 fps) than blood flow (25 fps), the sampling frequency still fulfilled the Shannon-Nyquist-Theorem (f_sample_ ≥ 2f_max_), allowing reliable assessment of cardiac contributions to CSF flow. Heart rates in our cohort ranged from 51 to 109 beats per minute (BPM; Table 1; Suppl Fig. 3), providing sufficient variation to examine cardiac-driven CSF dynamics. Data processing of the flow parameters, the r/c index was carried out using Matlab (Mathworks, Massachusetts, USA).

**Table 1:** Quantitative CSF and blood flow parameters, r/c indices, aqueduct area BPM and BF before (=7) and after treatment (*n* = 7) of AF patients.

	Treatment	Median	Significant	*p* value
Oscillatory CSF Volume in ml min^−1^	Before	1.21	*p* ≤ 0.05	0.008
	After	1.74		
CSF r/c Index	Before	1.40	*p* ≤ 0.05	0.039
	After	0.41		
Aqueduct Area in mm^2^	Before	3.94	NS	0.437
	After	5.06		
Peak CSF-Velocity in cm s^−1^	Before	2.94	p ≤ 0.05	0.016
	After	3.77		
JV Volume in ml min^−1^	Before	−255	NS	0.406
	After	−251		
JV r/c Index	Before	1.7	*p* ≤ 0.05	0.016
	After	0.5		
ICA Volume in ml min^−1^	Before	230	*p* ≤ 0.05	0.039
	After	338		
ICA r/c Index	Before	0.66	*p* ≤ 0.05	0.008
	After	0.27		
S. Sinus Volume in ml min^−1^	Before	−201	NS	0.179
	After	−245		
S. Sinus r/c Index	Before	1.5	p ≤ 0.05	0.039
	After	0.6		
BPM	Before	77.1	p ≤ 0.05	0.008
	After	58		
BF	Before	17.9	NS	0.078
	After	15.5		

Statistics based on Wilcoxon signed-rank test. Statistical significance was accepted at *p* ≤ 0.05.

The volumetric analysis of the individual brain regions was carried out using Freesurfer (surfer.nmr.mgh.harvard.edu, version 7.4.1). Based on the 3D T1-weighted dataset, automated brain segmentation was performed using the standard settings of the recon-all pipeline, resulting in each voxel being assigned to a specific anatomical structure.

Diffusion tensor images were generated using the FSL software suite (FMRIB, Oxford, UK, version 6.0.4). EPI data were corrected for eddy currents and minor head motion using FSL's “topup” and “eddy” tools (Andersson et al., 2003). From the processed data, voxel-wise maps of fractional anisotropy and diffusivity along the x-, y-, and z-axes were obtained.

Regions of interest (ROIs) for the DTI–ALPS analysis (Taoka et al., 2017) were manually defined on color-coded maps of principal diffusion directions using FIJI's freehand tool (open source FIJI software (Schindelin et al., 2012). ROIs were placed near the upper lateral ventricle region along projection and association fibers, where deep medullary veins run predominantly perpendicular to the imaging plane. Diffusivity values along each axis were extracted from these ROIs to calculate the ALPS index, defined as the ratio of diffusivity perpendicular to dominant fibers: specifically, the mean x-axis diffusivity in projection fibers and x-axis diffusivity in association fibers divided by the mean y-axis diffusivity in projection fibers and z-axis diffusivity in association fibers. For more details, see, Taoka et al. (Taoka et al., 2017).

T1 relaxation times were extracted from the white matter (WM) region at the level of the upper lateral ventricles, encompassing crossing projection and association fibers. ROI definition and tissue segmentation were performed using the FIJI/ImageJ plugin for ROI analysis (Schindelin et al., 2012). A non-parametric Wilcoxon signed-rank tests were applied for the datasets described above. Statistical significance was accepted at *p* values ≤ 0.05. All statistical analyses were carried out using GraphPad Prism 9 program (GraphPad Software, Inc).

Qualitative analysis of CSF dynamics was performed on mean b = 500 s mm^−2^ STEAM diffusion-weighted images using a previously established scoring method (Taoka et al., 2019). Signal intensity within the ventricles was compared to the surrounding white matter and rated as follows: 0 = no signal, 1 = lower than white matter, 2 = equal to white matter, 3 = higher than white matter. To calculate the scoring results, Fisher's exact test was used as described by Taoka et al. (Taoka et al., 2019).

Data handling for all datasets, in particular the computationally intensive volumetric analyses and the calculation of EPI-based DTI maps, was facilitated by components of the high-performance computing (HPC) research data management system (RDMS) previously established within the HeartAndBrain research project of the Department of Neurology, University Medical Center Göttingen (Telezki et al., 2024). Using this RDMS ensured consistent processing across all subjects and enabled reproducible execution of the computational steps involved in the multimodal MRI analysis.

### 2.4 Analysis of the physiological data

The physiological data were processed and analyzed offline in Python (https://www.python.org) (Bader et al., 2025). The signal curve of the breathing belt was smoothened with a Savitzky-Golay filter to remove artifacts. The same library was used to detect minima and maxima in the signal curve, representing the start positions of inhalation and exhalation, respectively. The four leads of the ECG data were z-score-normalized. This includes subtracting the mean value and dividing by the standard deviation. Additionally, the contained gradient-induced noise, caused by the MRI measurement was reduced by a convolutional filter. The noise frequency was determined by fast Fourier transformation. Subsequently, a lead with a pronounced cardiac component and adequate signal-to-noise ratio was selected manually to apply two peak detection algorithms from the NeuroKit2 library to reliably detect the ECG peaks. The raw pulse oximetry signal was directly used to apply a peak detection algorithm from the same library. The mean pulsation rates were calculated from temporal peak-to-peak distances that were averaged for the entire signal length of the corresponding modality data.

ECG and pulse oximetry (PPG) recordings were primarily collected for physiological monitoring and to provide additional characterization of the heart rhythm during MRI. Due to MRI-induced signal noise, the limited duration of the recordings, and the current lack of fully validated automated methods for peak detection under these conditions, a detailed analysis was not the focus of the present study. Nevertheless, the simultaneously recorded physiological signals were examined to obtain a general impression of cardiac rhythm dynamics and were used as supportive reference information during the interpretation of the CSF flow analyses and associated cardiac spectral components. This approach provided additional physiological context for the identification of heart peaks in the cerebrospinal fluid spectra.

## 3 Results

### 3.1 Patient's characteristics

Seven patients with atrial fibrillation participated in this study. The mean age was 66 years (SD ± 9.9) and six were male and one female. The cohort consisted of patients with either persistent (*n* = 4) or paroxysmal atrial fibrillation (*n* = 3). Six patients underwent interventional rhythm control therapy: three received electrical cardioversion (Subjects #1, #3, #4, Suppl Table 1), three underwent pulmonary vein isolation (Subjects #2, #5, #7, Suppl Table 1), and one underwent ablation of the cavotricuspid isthmus (Subject #6, Suppl Table 1). During the MRI session, six patients exhibited ongoing atrial fibrillation, whereas only one patient (Subject #7, Suppl Table 1) was in sinus rhythm at the time of image acquisition, indicating the absence of atrial fibrillation during the measurement. Subject #7 was documented to be in sinus rhythm during the pre-treatment MRI session, which represents a notable deviation from the expected pre-treatment physiological state. Accordingly, the pre-post comparison in this subject does not reflect a transition from AF to sinus rhythm and should be interpreted as a within-subject repeat measurement rather than a treatment-related change. The contribution of Subject #7 to group-level differences should therefore be interpreted with additional caution.

Several patients presented with cardiovascular risk factors, including type 2 diabetes (Subject #6), hypertension (Subjects #1, #2, #5), dyslipidemia (Subjects #1, #2, #3, #6, #7), and smoking (Subjects #1 and #6). All patients with AF were on oral anticoagulation and beta-blocker medication. The mean t-MoCA was 20.29 (± 2.1), indicating normal cognition performance in the participants.

### 3.2 CSF and blood flow analysis

**Fig. 2:**
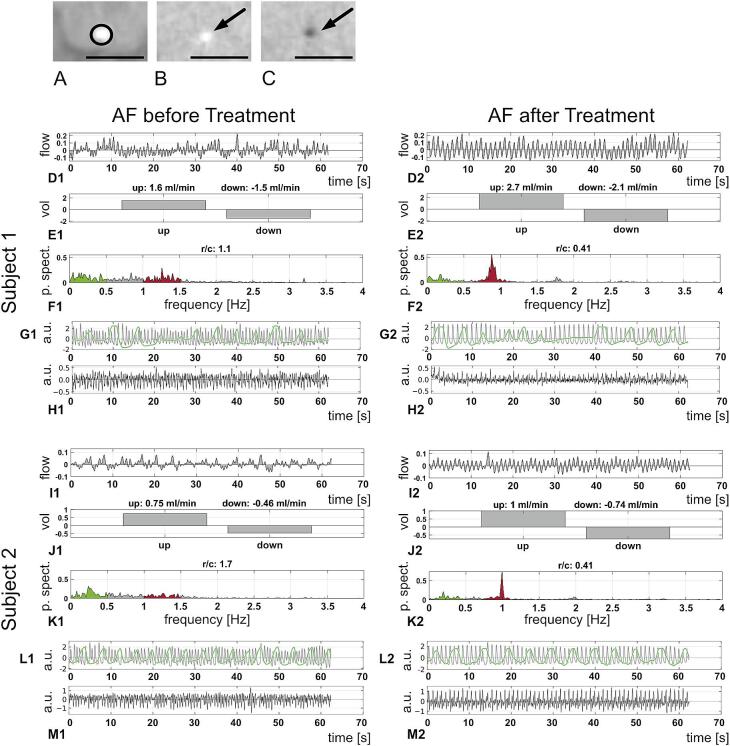
CSF flow plots in the aqueduct and the physiological plots in two subject #1 and #2 before (D1-M1) and after treatment (D2-M2). Subjects #1 and #2 were selected as representative examples because their measurements closely matched the cohort median. The measurement position corresponds to position 3 of Fig. 1. A–C show the region of interest in the aqueduct (circle in A) as magnitude image (A) and as phase contrast map of the upward flow (cranial; B arrow) and downward flow (caudal; C arrow). Subject 1: Evaluation of CSF flow dynamics in an AF patient before and after treatment. D1: CSF flow waveform before treatment. E1: Calculated cranial flow (1.6 ml min^−1^) and caudal flow (−1.5 ml min^−1^). F1: Power spectrum showing a high r/c index (1.1). G1–H1: Irregular heartbeat reflected in the pulse signal (G1, breathing curve overlaid in green; BF per min: 11.74) and ECG (H1). After treatment: D2: CSF flow waveform. E2: Increased cranial (2.7 ml min^−1^) and caudal (−2.1 ml min^−1^) flow. F2: Power spectrum with a reduced r/c index (0.41). G2–H2: Regular pulse waveform (G2) and ECG (H2). The breathing pattern appears more regular and relaxed (green curve in G2; BF per min: 11.68). Subject 2: I1: CSF flow waveform before treatment. J1: Cranial flow (0.75 ml min^−1^) and caudal flow (−0.46 ml min^−1^). K1: Power spectrum showing a high r/c index (1.7). L1–M1: Irregular pulse waveform (L1, breathing curve in green; BF per min: 15.51) and ECG (M1). After treatment: I2: CSF flow waveform. J2: Increased cranial (1.0 ml min^−1^) and caudal (−0.74 ml min^−1^) flow compared with pre-treatment. K2: Power spectrum with a reduced r/c index (0.41). L2–M2: Regular pulse waveform and ECG. Breathing appears more regular and relaxed (green curve in L2, BF per min: 11.14). Scale bars 2 cm. Pulse and ECG signals are shown in arbitrary units (a.u.). Flow is given in ml s^−1^, volume in ml min^−1^. Power spectra illustrate dominant frequency components, with higher frequencies assigned to cardiac (red) and lower frequencies to respiratory contributions (green). a.u. = arbitrary units.

**Fig. 3:**
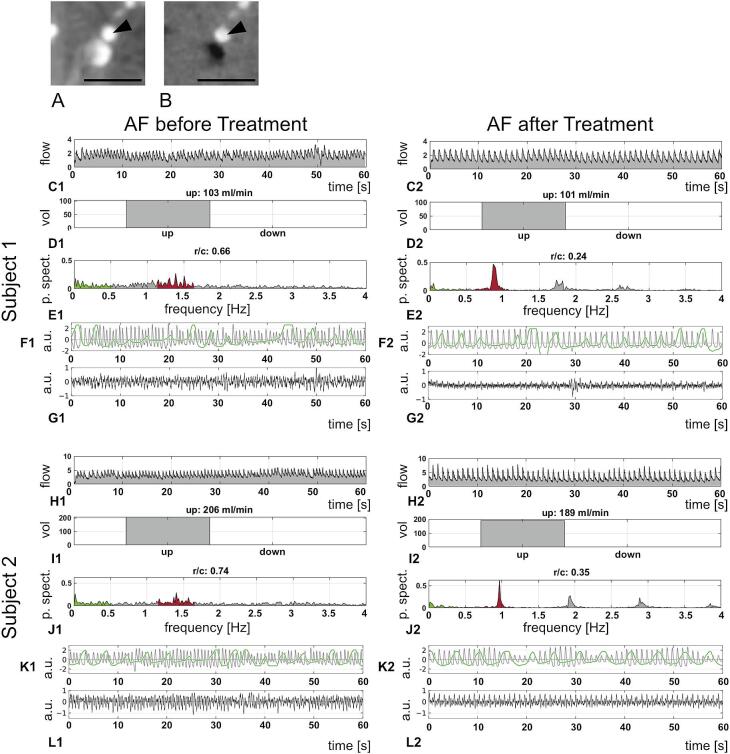
Blood flow plots of the ICA in subject #1 and #2 before (C1-L1) and after treatment (C2-L2). The measurement position corresponds to position 1 of Fig. 1. A, B show the region of interest in the ICA (arrowhead in A) and as phase contrast map of the upward flow (arrowhead in A). Subject 1: C1–C2: ICA flow waveforms before and after treatment. D1: Cranial flow volume before treatment (103 ml min^−1^). D2: Cranial flow volume after treatment (101 ml min^−1^). E1–E2: r/c index decreased from 0.96 to 0.24. F1–F2: Pulse waveform before and after treatment (breathing overlaid in green). G1–G2: ECG before and after treatment. Subject 2: H1–H2: ICA flow waveforms. I1: Cranial flow before treatment (206 ml min^−1^). I2: Cranial flow after treatment (189 ml min^−1^). J1–J2: r/c index decreased from 0.74 to 0.35 after treatment. K1–K2: Pulse waveform before and after treatment (breathing in green). L1–L2: ECG before and after treatment. Scale bars 2 cm. Flow is given in ml s^−1^, volume in ml min^−1^. Cardiac (red) and respiratory (green) frequency components are shown in the spectra. a.u. = arbitrary units.

**Fig. 4:**
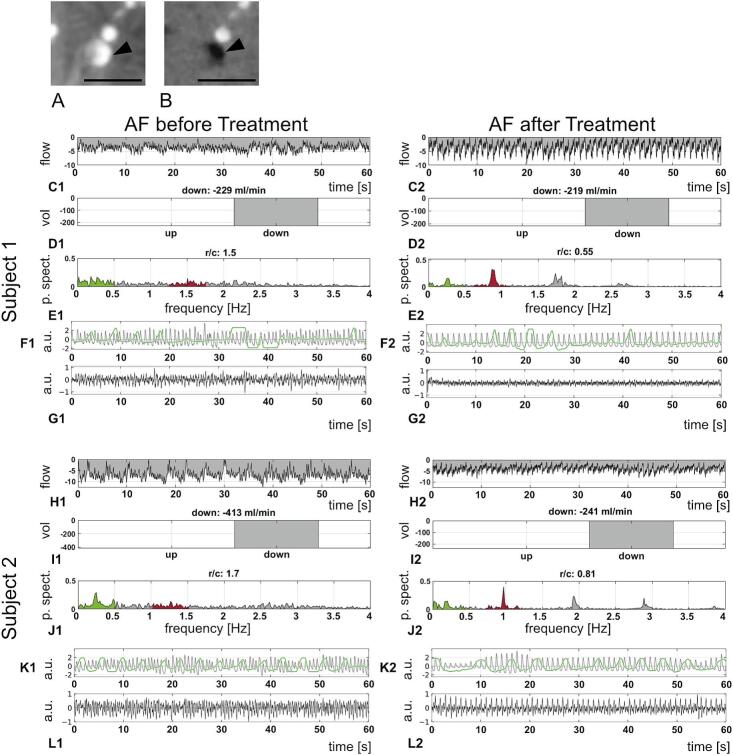
Blood flow plots of the JV in subject #1 and #2 before (C1-L1) and after treatment (C2-L2). The measurement position corresponds to position 2 of Fig. 1. A, B show the region of interest in the JV (arrowhead in A) and as phase contrast map of the downward flow (arrowhead in A). Subject 1: C1–C2: JV flow waveforms before and after treatment. D1: Caudal flow volume before treatment (−229 ml min^−1^). D2: Caudal flow volume after treatment (−219 ml min^−1^). E1–E2: r/c index decreased from 1.5 to 0.55 after treatment. F1–F2: Pulse waveform before and after treatment (breathing in green). G1–G2: ECG before and after treatment. Subject 2: H1–H2: JV flow waveforms. I1: Caudal flow before treatment (−413 ml min^−1^). I2: Caudal flow after treatment (−241 ml min^−1^). J1–J2: r/c index decreased from 1.7 to 0.81 after treatment. K1–K2: Pulse waveform before and after treatment (breathing in green). L1–L2: ECG before and after treatment. Scale bars 2 cm. Flow is given in ml s^−1^, volume in ml min^−1^. Spectra show cardiac (red) and respiratory (green) components. a.u. = arbitrary units.

**Fig. 5:**
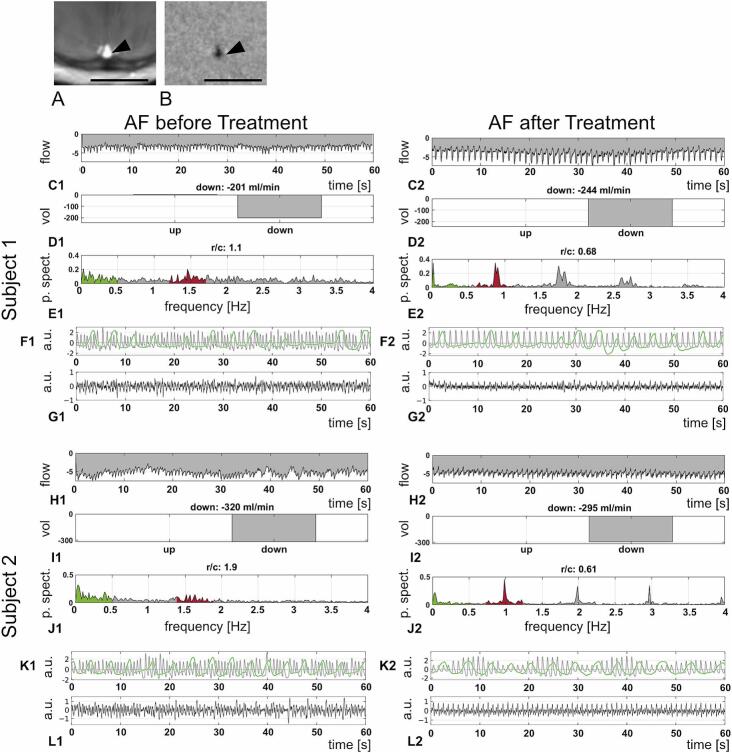
Blood flow plots of the sagittal sinus in subject #1 and #2 before (C1-L1) and after treatment (C2-L2). The measurement position corresponds to position 4 of Fig. 1. A, B show the region of interest in the sagittal sinus (arrowhead in A) and as phase contrast map of the downward flow (arrowhead in A). Subject 1: C1–C2: Sagittal sinus flow waveforms before and after treatment. D1: Caudal flow volume before treatment (−201 ml min^−1^). D2: Caudal flow volume after treatment (−244 ml min^−1^). E1–E2: r/c index decreased from 1.1 to 0.68 after treatment. F1–F2: Pulse waveform before and after treatment (breathing in green). G1–G2: ECG before and after treatment. Subject 2: H1–H2: Sagittal sinus flow waveforms. I1: Caudal flow before treatment (−320 ml min^−1^). I2: Caudal flow after treatment (−295 ml min^−1^). J1–J2: r/c index decreased from 1.9 to 0.61 after treatment. K1–K2: Pulse waveform before and after treatment (breathing in green). L1–L2: ECG before and after treatment. Scale bars 2 cm. Flow is given in ml s^−1^, volume in ml min^−1^. Power spectra illustrate cardiac (red) and respiratory (green) frequency components. a.u. = arbitrary units.

**Supplementary Fig S1:**
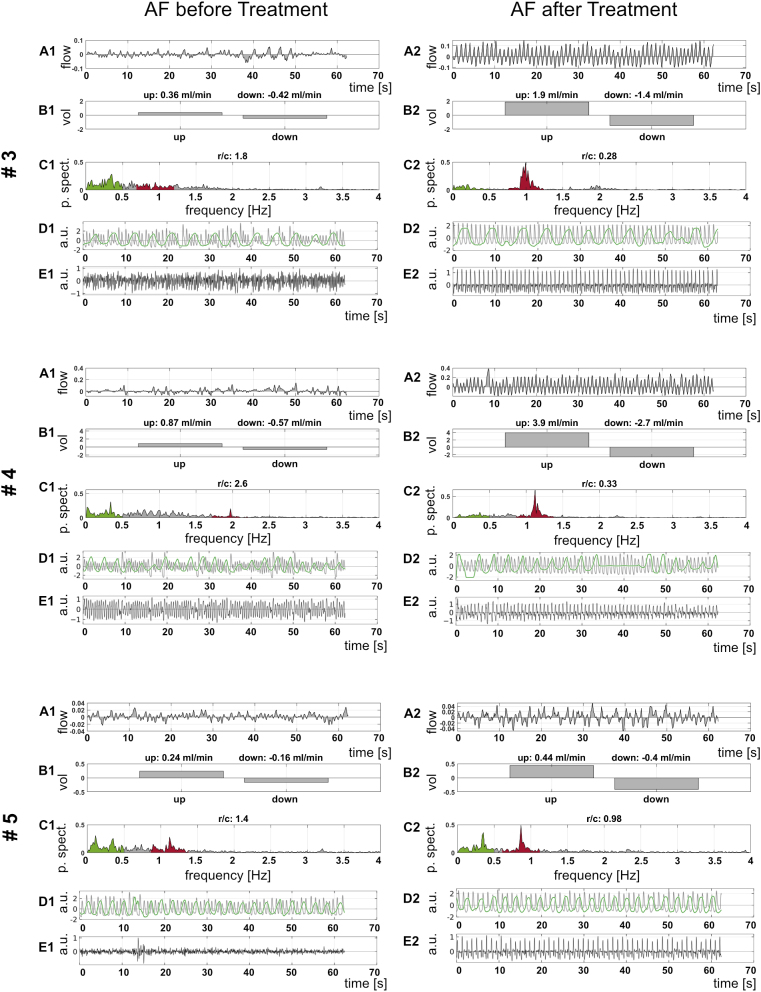
Evaluation of CSF flow dynamics in AF patient #3 - #5 before (A1-E1) and after treatment (A2-E2). #3:. A1-A2: CSF flow waveform. B1: Calculated cranial flow (0.36 ml min^−1^) and caudal flow (−0.42 ml min^−1^). C1: Power spectrum showing a high r/c index (1.8). D1–E1: Pulse signal (D1, breathing curve overlaid in green) and ECG (E1). B2: Calculated cranial flow (1.9 ml min^−1^) and caudal flow (−1.4 ml min^−1^). C2: Power spectrum lower r/c index (0.28). D2–E2: Pulse signal (D2, breathing curve overlaid in green) and ECG (E2). #4: A1-A2: CSF flow waveform. B1: Calculated cranial flow (0.87 ml min^−1^) and caudal flow (−0.57 ml min^−1^). C1: Power spectrum showing a high r/c index (2.6). D1–E1: Pulse signal (D1, breathing curve overlaid in green) and ECG (E1). B2: Calculated cranial flow (3.9 ml min^−1^) and caudal flow (−2.7 ml min^−1^). C2: Power spectrum lower r/c index (0.33). D2–E2: Pulse signal (D2, breathing curve overlaid in green) and ECG (E2). #5: A1-A2: CSF flow waveform. B1: Calculated cranial flow (0.24 ml min^−1^) and caudal flow (−0.16 ml min^−1^). C1: Power spectrum showing a high r/c index (1.4). D1–E1: Pulse signal (D1, breathing curve overlaid in green) and ECG (E1). B2: Calculated cranial flow (0.44 ml min^−1^) and caudal flow (−0.4 ml min^−1^). C2: Power spectrum lower r/c index (0.98). D2–E2: Pulse signal (D2, breathing curve overlaid in green) and ECG (E2).

**Supplementary Fig S2:**
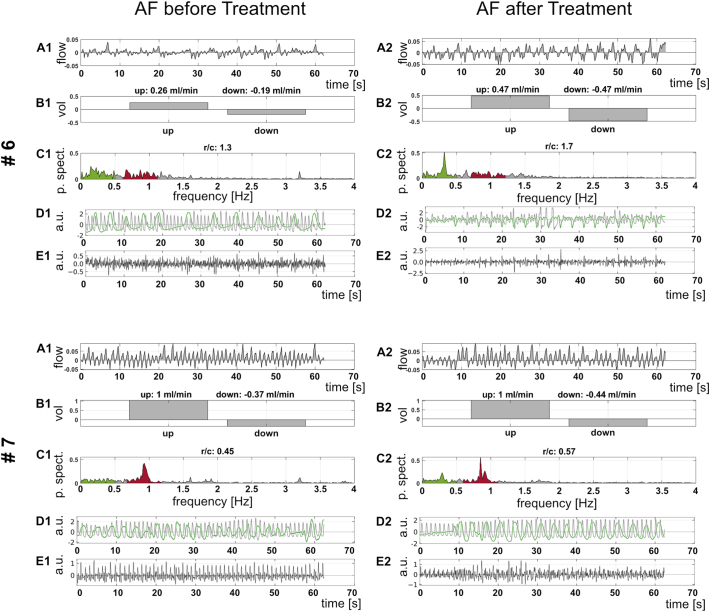
Evaluation of CSF flow dynamics in AF patient #6 - #7 before (A1-E1) and after treatment (A2-E2). #6:A1-A2: CSF flow waveform. B1: Calculated cranial flow (0.26 ml min^−1^) and caudal flow (−0.19 ml min^−1^). C1: Power spectrum showing the r/c index (1.3). D1–E1: Pulse signal (D1, breathing curve overlaid in green) and ECG (E1). B2: Calculated cranial flow (0.47 ml min^−1^) and caudal flow (−0.47 ml min^−1^). C2: Power spectrum and the r/c index (1.7). D2–E2: Pulse signal (D2, breathing curve overlaid in green) and ECG (E2). #7: A1-A2: CSF flow waveform. B1: Calculated cranial flow (1 ml min^−1^) and caudal flow (−0.37 ml min^−1^). C1: Power spectrum and the r/c index (0.45). D1–E1: Pulse signal (D1, breathing curve overlaid in green) and ECG (E1). B2: Calculated cranial flow (1 ml min^−1^) and caudal flow (−0.44 ml min^−1^). C2: Power spectrum and the r/c index (0.57). D2–E2: Pulse signal (D2, breathing curve overlaid in green) and ECG (E2). Flow is given in ml s^−1^, volume in ml min^−1^. Power spectra illustrate cardiac (red) and respiratory (green) frequency components. a.u. = arbitrary units.

**Supplementary Fig S3:**
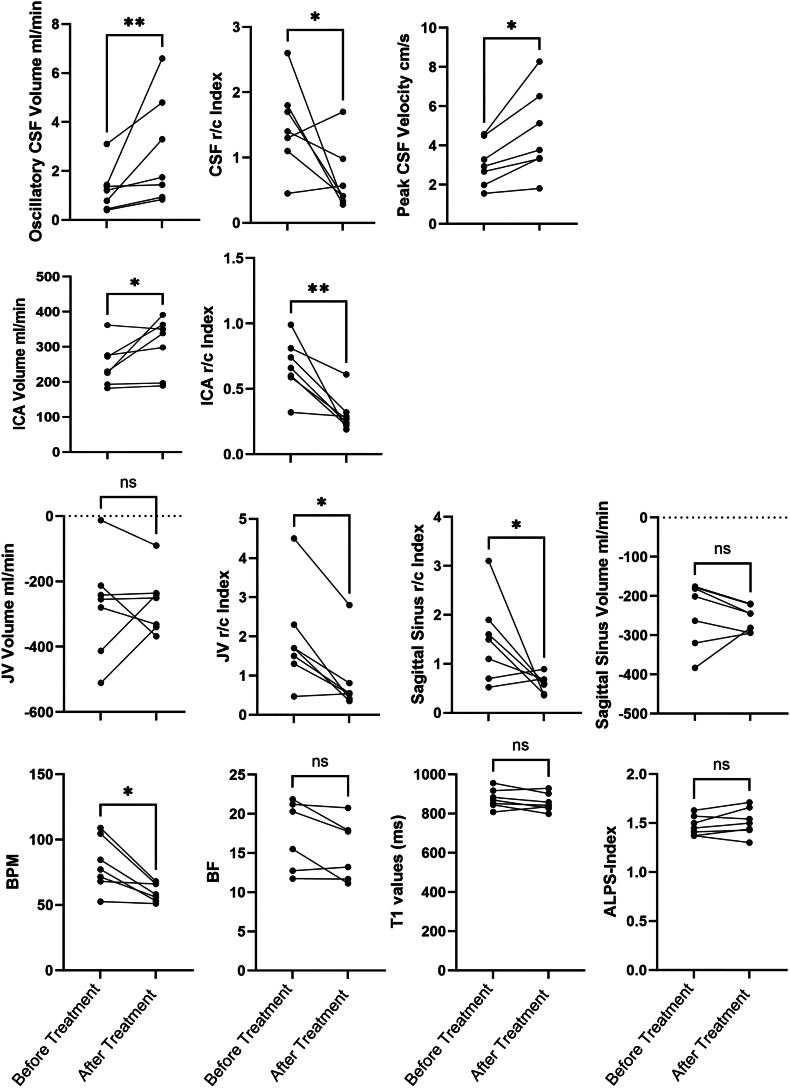
Plots of aqueductal oscillatory CSF flow volumes, ICA, JV, and sagittal sinus flow volumes, r/c indices, peak CSF velocity, beats per minute (BPM), breathing frequency (BF), T1 values, and the ALPS index in 7 patients before and after individualized treatment. Oscillatory CSF- and blood volumes calculated over the 60 s acquisition time period. CSF: cerebrospinal fluid; ICA: internal carotid artery; JV: jugular vein. Wilcoxon signed-rank test was applied. Statistical significance was accepted at *p* values *<*0.05. * p value <0.05, ** p value *<*0.01; ns = not significant.

We used real-time phase-contrast flow MRI in areas of the aqueduct, the ICA, the JV and the sagittal sinus (Fig. 1) to capture the CSF- and blood flow patterns, as well as corresponding flow volume (Fig. 2, Fig. 3, Fig. 4, Fig. 5). Spectral analysis of the CSF flow in the aqueduct showed both respiratory (low-frequency) components and cardiac (high-frequency) components, expressed as the respiratory/cardiac ratio or r/c index (Yildiz et al., 2017). Cardiac arrhythmicity was associated with altered CSF and blood flow patterns (Fig. 2, Fig. 3, Fig. 4, Fig. 5, Suppl. Fig.1,2,3; Table 1).

Before treatment, AF patients had lower CSF flow (CSF flow 1.21 ml min^−1^; Table 1) and higher r/c indices (CSF r/c index, 1.4; Table 1; Suppl Fig. 3) in contrast to CSF flow values after individual treatment (CSF flow 1.74 ml min^−1^; CSF r/c index, 0.41; Table 1). The size of the aqueduct did not change after treatment, but the peak CSF velocities increased from 2.94 cm s^−1^ to 3.77 cm s^−1^ (Fig. 2, Table 1), resulting in a higher CSF flow (CSF flow 1.74 ml min^−1^, Table 1; Suppl Fig. 3). Throughout the manuscript, increased CSF flow is referred to as more pronounced CSF oscillation or CSF dynamics.

As shown in Suppl. Fig. 1–2, CSF flow dynamics were higher after treatment in all patients and were consistently observed at the individual level. Subject-specific variations were present and may have related to individual clinical conditions, including the absence of AF during pre-treatment scanning (Suppl. Fig. 2, #7) or marked post-cardioversion physiological changes (Suppl. Fig. 2, #6). Subject #6 experienced marked weight gain between the two MRI acquisitions and exhibited a relatively stronger respiratory contribution to CSF dynamics compared to the remainder of the cohort. These individual characteristics may have accounted for part of the observed variability and were documented in Suppl. Table S1 and Suppl. Fig. 1–2.

For blood flow in the ICA, JV and the sagittal sinus the r/c index decreased after treatment (Fig. 3, Fig. 4, Fig. 5, Table 1), and the arrhythmic flow pattern changed to periodic rhythmic pattern dominantly modulated by the regular heartbeat. Interestingly, the ICA flow slightly increased after treatment (ICA flow before treatment 230 ml min^−1^ to 388 ml min^−1^ after treatment; Table 1), whereas the JV volume remain unchanged and sagittal sinus blood volume show a trend toward higher flow values (Table 1). In some spectra, a minor peak above 2 Hz was observed in addition to the dominant physiological components. Cardiac fundamental frequencies in this cohort ranged from approximately 0.85 to 1.8 Hz, a peak around 2 Hz and 4 Hz would correspond to the harmonic of the cardiac waveform, which is plausible for non-sinusoidal pulsatile flow signals. Higher harmonics of pulsatile vascular waveforms may occur; however, because spectral components above 4 Hz were not considered in the present analysis. Reconstruction-related artifacts or noise remain possible alternative explanations but appear less likely given the consistency of this feature across subjects.

The BPM, calculated via the CaFuR software, decreased after individual treatment (77.1 BPM to 58 BPM; Table 1, Suppl Fig. 3) of AF. The calculated breathing frequency (BF; Table 1) decreased slightly in individual subjects (Fig. 2; subject 1: BF per min before treatment 11.74 and BF after treatment 11.68; subject 2: BF per min before treatment 15.51 and BF after treatment 11.14) but did not show significant group-level differences.

We further used the ECG and PPG data, originally intended for monitoring purposes only, to also access the regularity of heartbeats during the temporally low RT-PC CSF measurement. Since the ECG data contains much MRI-induced noise, and the more reliable PPG signal is still influenced by finger movements, only together they provide a basis for heartbeat regularity evaluation. In all subjects (Table 2), except for subject #6, the median duration between two consecutive pulse peaks was increased after the AF treatment (median PPG before: 0.80 s, after: 1.01 s) indicating a reduced heart rate and supporting the results evaluated on RT-PC flow data via CaFuR software. Additionally, the interquartile range (Q1-Q3) based on all PPG peak-to-peak-durations was reduced from 0.20 s to 0.10 s. The ECG-derived interquartile range is exceptionally increased in subject #6 and #7, representing the already mentioned subject-specific characteristics of an increased body weight (subject #6) and the missing atrial fibrillation in the baseline measurement (subject #7).

**Table 2:** Interquartile range (Q1–Q3) of PPG- and ECG-derived peak-to-peak intervals before and after treatment.

Subject	PPG before	PPG after	ECG before	ECG after
1	0.17 (s)	0.03 (s)	0.31 (s)	0.08 (s)
2	0.20 (s)	0.02 (s)	0.25 (s)	0.02 (s)
3	0.30 (s)	0.05 (s)	0.35 (s)	0.05 (s)
4	0.05 (s)	0.07 (s)	0.53 (s)	0.07 (s)
5	0.21 (s)	0.05 (s)	0.60 (s)	0.04 (s)
6	0.46 (s)	0.40 (s)	0.39 (s)	0.58 (s)
7	0.04 (s)	0.05 (s)	0.04 (s)	0.17 (s)

**Fig. 6:**
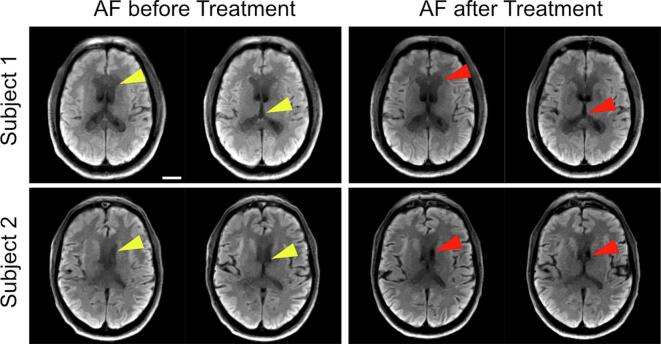
Diffusion-weighted imaging (DWI) b = 500 s/mm^2^ in subject #1 and #2 before and after treatment. At b = 500 s/mm^2^, the signal suppression is incomplete, potentially providing insights into CSF dynamics within the ventricular system. Signal voids in the ventricles (yellow arrowheads, before treatment) are more prominent after treatment (indicated by red arrowheads). Scale bar 2 cm.

To assess whole-brain CSF motion, we applied the method proposed by Taoka et al. (Taoka et al., 2019). Here we analyzed mean DWI data in the ventricles before and after treatment. On the b = 500 s mm^−2^ DWI, we evaluated the presence and degree of signal void, representing the CSF motion, in the anterior horn, the body, atrium, temporal horn, 3rd ventricle and 4th ventricle (Fig. 6, Table 3). After treatment, the CSF filled region of the anterior horn showed slightly higher flow-related signal alterations (Fig. 6, Table 3).

**Table 3:** Comparison of scores for CSF signal intensity or signal void based on DWI data before (n = 7) and after (n = 7) treatment of AF patients.

	Score	0	1	2	3	Significant	*p* value
Anterior horn	Before	0	1	6	0	*p* ≤ 0.05	0.029
	After	1	5	1	0		
Body	Before	0	7	0	0	NS	0.461
	After	2	5	0	0		
Atrium	Before	0	7	0	0	NS	0.192
	After	4	4	0	0		
Temporal horn	Before	0	2	5	0	NS	0.999
	After	0	2	5	0		
3rd ventricle	Before	2	5	0	0	NS	0.592
	After	4	3	0	0		
4th ventricle	Before	0	7	0	0	NS	0.192
	After	3	4	0	0		

For statistics, Fisher's exact test was used. Statistical significance was accepted at p values ≤ 0.05.

### 3.3 DTI-ALPS index and surrounding tissue

We evaluated glymphatic efflux-related properties using the diffusion-based DTI-ALPS index, first proposed by Taoka and colleagues (Taoka et al., 2017). In numerous studies (Lee et al., 2022; Steward et al., 2021; Wu et al., 2024; Xiong et al., 2024), this index has been used as a surrogate marker for the efflux pathway of the GS; therefore, it has been incorporated into the current study design. In the present cohort, DTI-ALPS values did not differ before and after treatment (Table 4). To increase confidence that observed changes in the flow dynamics or the ALPS-Index reflect genuine physiological effects rather than structural variability, we included complementary quantitative parameters derived by T1 maps. To assess tissue integrity of the white matter and pathological alterations not only in areas directly relevant for the DTI-ALPS index calculations, but also in adjacent regions that could impact the resulting DTI-ALPS index values, we conducted a thorough evaluation using a single quantitative map of the T1 relaxation times at the corresponding slice positions. ROI analysis showed no differences in T1-relaxation times in the WM before and after treatment (Table 4).

**Table 4:** Comparison of the DTI-ALPS-Index and T1 values (in ms) before (n = 7) and after (n = 7) of AF treatment.

	Treatment	Median	Significant	*p* value
ALPS-Index	Before	1.45	NS	0.234
	After	1.50		
T1-relaxation time	Before	869	NS	0.218
	After	842		

Statistics based on Wilcoxon signed-rank test. Statistical significance was accepted at p ≤ 0.05.

Automatic whole brain segmentation and volumetric analysis revealed no significant differences in brain regions, including whole brain, white matter, and ventricles before and after treatment of AF (Table 5). The whole-brain analysis of the ventricles and white matter corroborated the quantitative findings from the T1 maps as well as from the DWI measurements, represented by the DTI-ALPS index.

**Table 5:** Volumes (in mm^3^) of selected structure based on T1 weighted before (=7) and after treatment (n = 7) of AF patients.

	Treatment	Median	Significant	*p* value
Left lateral ventricle	Before	16,040	NS	0.109
	After	16,273		
Right lateral ventricle	Before	15,617	NS	0.297
	After	15,411		
3rd ventricle	Before	2189	NS	0.578
	After	2261		
4th ventricle	Before	2057	NS	0.218
	After	1905		
Whole brain	Before	1,203,262	NS	0.218
	After	1,192,893		
WM	Before	494,172	NS	0.375
	After	472,756		

Statistics based on Wilcoxon signed-rank test. Statistical significance was accepted at p ≤ 0.05.

## 4 Discussion

This exploratory study investigated vascular and oscillatory CSF dynamics relevant to neurofluid physiology in patients with atrial fibrillation before and after treatment using a fast multimodal MRI protocol. The present investigation represented an exploratory pre–post cohort without a sham or matched control condition. Therefore, the observed alterations cannot be attributed causally to the intervention itself and should be interpreted as within-subject physiological associations. The primary aim was descriptive characterization of flow dynamics rather than demonstration of therapeutic efficacy. As discussed recently (Kamagata et al., 2023; Taoka et al., 2024), it is essential to employ a combination of techniques in order to gain insight into the perivascular fluid transport. The multimodal MRI methods used in the present study allowed assessment of vascular and CSF oscillatory dynamics, flow waveform characteristics, and structural brain measures within the same examination.

Cardiac- and low frequency -driven vascular and CSF oscillations are considered important contributors to neurofluid dynamics and perivascular fluid transport (Alperin and Burman, 2024; Burman and Alperin, 2024; Nedergaard, 2013; Saka and Topçuoğlu, 2023). Despite growing interest in this field, the precise mechanisms and driving forces of flow dynamic physiology in humans remain incompletely understood and continue to be debated (Liu et al., 2025; Rasmussen et al., 2022). Recent findings suggest that irregular and aperiodic blood flow patterns associated with AF may contribute to altered vascular physiology and cognitive decline (Guo et al., 2025). In this context, irregular blood flow and microembolic events, such as those associated with AF, may contribute to cerebral microvascular damage, thereby alter neurofluid dynamics (Blum and Conen, 2023a; Trumbore, 2021).

Current physiological models propose dynamic interactions between arterial pulsation, venous outflow, respiration, and CSF oscillations, although these relationships cannot be directly resolved by the present measurements (Burman and Alperin, 2024; Kollmeier et al., 2022a).

In our study, the observed increase in ICA flow was not accompanied by obvious proportional changes in venous flow and is therefore not straightforward to interpret, and the unilateral assessment of the internal jugular vein in some subjects further limits direct comparison of venous flow volumes across individuals. Cerebral venous drainage is distributed across multiple pathways, including the internal jugular veins, vertebral venous plexus, epidural veins, and collateral vessels, which were not fully captured in the present study. The present data do not permit a complete assessment of intracranial flow balance. One plausible interpretation is that the observed ICA changes reflect altered pulsatility flow dynamics rather than a proportional increase in global cerebral blood flow.

In a recent study using a heart failure mouse model with reduced ejection fraction, the authors reported altered brain fluid dynamics together with reduced cerebral blood flow and impaired perivascular fluid transport (Kritsilis et al., 2025). However, the extent to which these findings translate to human physiology requires further investigation.

AF alters not only blood flow dynamics, (Blum and Conen, 2023a, Blum and Conen, 2023b), but also CSF dynamics, which has previously been ignored. Previous work showed that AF reduces CSF dynamics in ventricular regions and disrupts aqueductal CSF oscillations (Hofer et al., 2025). Although different treatment modalities were applied in this cohort (electrical cardioversion or pulmonary vein isolation), the physiological outcomes converged toward the same pattern. The study was not powered to compare subgroups statistically, and no qualitative differences in CSF or blood flow outcomes between treatment modalities were observed. The observed post-treatment changes were consistent with restoration of rhythmic cardiac activity at the time of measurement, rather than the specific interventional technique. We acknowledge that during AF, broader and less stationary cardiac spectral components may extend beyond the predefined bandwidth and therefore may not be fully captured. This may contribute to an underestimation of the cardiac component before treatment and thereby influence the r/c ratio. Accordingly, the r/c index should be interpreted as a surrogate marker reflecting the relative waveform composition and the balance between low-frequency and cardiac-frequency components.

While restoration of sinus rhythm was associated with more regular and periodic cardiac-driven CSF flow, the observed increase in CSF flow was not fully explained by periodicity alone. One plausible interpretation is that irregular cardiac activity produces less coherent pulsatile flow patterns, which may alter the transmission of vascular pulsations to the CSF system. Restoration of a more regular cardiac rhythm may therefore result in more periodic CSF oscillatory patterns. However, this interpretation remains speculative and cannot be directly resolved within the present study. The observed association between altered blood flow patterns and CSF flow supports a close physiological relationship between cardiovascular pulsatility and CSF motion, although the mechanisms underlying the observed changes remain unclear and require further investigation.

These quantitative changes occurred in the absence of detectable structural brain alterations, including stable ventricular volumes, unchanged white matter integrity, and unaltered DTI-ALPS indices.

Additional examination of the large ventricular spaces revealed slightly increased CSF movement in the anterior horn (Fig. 6; Table 3). These evaluations were based on mean DWI data using a simple grey scale difference method as proposed by Taoka and colleagues (Taoka et al., 2019), should therefore be interpreted with caution given the exploratory nature of the study.

In previous studies, the DTI-ALPS index was regarded as a surrogate marker for the efflux pathway via the perivascular space (Lee et al., 2022; Saito et al., 2023; Steward et al., 2021; Wu et al., 2024; Xie et al., 2024; Xiong et al., 2024) and an important parameter for this part of the GS function in AF (Guo et al., 2025). It has been suggested that the movement of fluids within the GS depends on rhythmic oscillations driven by the regular heartbeat. Disturbances in the artery pulsation rhythm, abnormal beating frequency, decreased arterial pulsatility during arrhythmia and reduced CSF oscillation in patients with AF may contribute to reduced perivascular flow, further leading to altered neurofluid dynamics.

As discussed previously, the DTI-ALPS index alone does not reflect all aspects of the GS (Georgiopoulos et al., 2024; Ringstad, 2024), (Barisano et al., 2021; Ringstad, 2024). Consequently, interpretations derived from the DTI-ALPS index should be approached with caution, acknowledging its potential limitations in resolving the complex dynamics of perivascular fluid flow. In our study, the calculated DTI-ALPS indices (Taoka et al., 2017) remained unchanged before and after treatment of AF (Table 4). Notably, all patients in our study were cognitively normal and did not display detectable structural brain alterations. This distinguishes our cohort from recent work linking AF to cognitive impairment and reduced ‘glymphatic’ efflux (Guo et al., 2025), and suggests that the effects observed here may capture an earlier, physiologically compensated stage.

Physiological recordings were acquired primarily for monitoring purposes. Although they provided supportive information regarding cardiac and respiratory influences, the current dataset does not allow comprehensive modeling of cardiorespiratory–CSF coupling networks as described in long-duration recordings (Bashan et al., 2012; Bartsch et al., 2015).

Whether hemodynamic alterations precede and contribute to later cognitive decline remains an open question. The present results describe short-term physiological alterations and do not establish prognostic relevance. Long-term observations following AF treatment could further elucidate the complex interplay between blood and CSF flow dynamics and fluid circulation, providing deeper insight into the functional mechanisms of the neurofluid dynamics.

## 5 Limitations

The main limitation of the present study is the small sample size, which restricts statistical power and precluded subgroup analyses, therefore the effect-size estimates should be interpreted with caution. In addition, absence of test–retest reproducibility assessment limits the ability to distinguish biological change from measurement variability. Statistical analyses were performed as within-subject comparisons across predefined physiological parameters and anatomical regions. Given the exploratory design, no formal correction for multiple comparisons was applied. Accordingly, the findings should be interpreted cautiously and considered hypothesis-generating.

The relatively long interval between pre- and post-treatment MRI (∼20 months) represents a major limitation of the study. During this period, changes in medication, body weight, and other clinical factors may have occurred and could have influenced the observed physiological parameters. Given the small sample size, these potential confounders could not be modeled or controlled for. The observed changes should be interpreted as within-subject changes rather than treatment-specific effects. Future studies with shorter follow-up intervals and controlled conditions will be required to isolate treatment-specific effects.

The CSF acquisition parameters reflect a trade-off between temporal resolution, spatial resolution, and signal-to-noise ratio. While the applied temporal resolution (8 fps) fulfills the Nyquist Theorem for the relevant physiological frequency range and allows reliable identification of cardiac and respiratory frequency components, it may underestimate peak velocities and oscillatory amplitudes. Specifically, it cannot be excluded that part of the observed post-treatment increase in CSF flow amplitude reflects reduced temporal underestimation under more regular cardiac conditions, rather than a purely physiological increase in CSF oscillatory volume. Cardiac cycles in AF are shorter and more irregular, potentially leading to greater undersampling error before treatment; after restoration of sinus rhythm, the longer and more regular cardiac cycle provides more samples per oscillatory period, potentially improving amplitude estimation. This alternative explanation cannot be fully excluded within the present study design.

One limitation of venous blood flow analysis is that flow in the JV could not be analyzed bilaterally in all participants. If one JV was hypoplastic or could not be reliably identified, only the contralateral side was measured. Consequently, JV flow values may not be directly comparable across participants. Furthermore, unilateral JV flow may represent either the majority or only a portion of the total jugular outflow and should therefore not be interpreted as a complete estimate of total cerebral venous outflow.

The present data are hypothesis-generating and are intended to inform future controlled longitudinal investigations. Future participants will undergo comprehensive cognitive assessments, alongside additional blood analyses to evaluate surrogate biomarkers indicative of perivascular fluid transport. Finally, although physiological recordings were collected, integrating them with the real-time MRI data to characterize cardiorespiratory interaction networks (von der Ohe et al., 2026) remains a methodological challenge for future work. Larger prospective studies incorporating cognitive testing, blood-based biomarkers, and extended multimodal flow analyses will be needed to establish clinical relevance.

## 6 Conclusion

Within-subject comparisons demonstrated that changes in cardiac rhythm were accompanied by measurable shifts in CSF and blood flow dynamics. Quantitative DTI-ALPS, brain volume analysis and T1 mapping data confirmed that white matter and overall brain structure remained unchanged. The observed alterations in vascular and CSF oscillatory flow volumes support a close association between cardiac rhythm and neurofluid dynamics. Further research in larger cohorts with longitudinal follow-up is warranted to better characterize the relationship between blood and CSF flow dynamics and complex fluid transport.

The following are the supplementary data related to this article.

Supplementary Material 4

## CRediT authorship contribution statement

**Sabine Hofer:** Writing – review & editing, Writing – original draft, Visualization, Validation, Supervision, Project administration, Methodology, Investigation, Formal analysis, Data curation, Conceptualization. **Marlena Schnieder:** Writing – original draft, Supervision, Investigation. **Leonie Polster:** Writing – review & editing, Writing – original draft, Validation, Formal analysis. **Angelika S. Bader:** Writing – review & editing, Writing – original draft, Formal analysis. **Vitali Telezki:** Writing – review & editing, Writing – original draft, Software, Formal analysis, Data curation. **Peter Dechent:** Writing – review & editing, Writing – original draft, Supervision, Data curation, Conceptualization. **Mathias Bähr:** Writing – review & editing, Writing – original draft, Project administration, Funding acquisition, Conceptualization.

## Declaration of competing interest

The authors declare that they have no known competing financial interests or personal relationships that could have appeared to influence the work reported in this paper.

## Data Availability

No data was used for the research described in the article.
