# Supplementary material for: Within-subject alterations in CSF and blood flow dynamics following rhythm-control intervention in atrial fibrillation: An exploratory multimodal MRI study
Source: Neuroimage Clin. 2026 Jul 3;51:104029. doi: 10.1016/j.nicl.2026.104029 (PMC13348055; doi:10.1016/j.nicl.2026.104029)
Supplement: Supplementary Material 4 [file mmc4.docx]

Table S1: Baseline characteristics and atrial fibrillation–related clinical parameters of the study subjects before and after intervention.

| **Subject** | **Age** | **Period between MRI** | **Weight** | | **BMI** | | **Medication** | | **Type of AF** | **Treatment** | **First diagnosed** |
| --- | --- | --- | --- | --- | --- | --- | --- | --- | --- | --- | --- |
|  |  |  | **Before** | **After** | **Before** | **After** | **Before** | **After** |  |  |  |
| 1 | 71 | 20 | 102 | 103 | 31.5 | 31.8 | Dabigatran, Furosemid, Bisoprolol, Ramipril | Furosemid, Bisoprolol, Ramipril | persistent | EKV | 2004 |
| 2 | 70 | 21 | 100 | 90 | 26.6 | 23.9 | Apixaban, Bisoprolol, Ramipril, Pantoprazol, Dapaglifozin, Tamsulosin | Apixaban, Ramipril, Forxiga, Amlodin, Tamsolusin | persistent | PVI | 2017 |
| 3 | 51 | 24 | 87 | 85 | 26.3 | 25.7 | Apixaban, Bisoprolol, Ramipril, Levothyroxin, Imbruvica | Zanubrutinib | persistent | EKV | 2022 |
| 4 | 82 | 24 | 79 | 76 | 27.7 | 25.7 | Apixaban, Verapamil, Flecainid, Montelukast, Tiotropium | Verapamil, Flecainid, Montelukast, Mometason | paroxysmal | EKV | 2008 |
| 5 | 58 | 24 | 93 | 95 | 28.7 | 29.3 | Apixaban, Bisoprolol, Candesartan, Amiodaron, Pantoprazol | Apixaban, Atorvastatin, Dapagliflozin, Amlodipin, Pantoprazol | persistent | PVI | 2022 |
| 6 | 63 | 21 | 87 | 100 | 29.4 | 33.8 | Rivaroxaban, Metoprolol, Atorvatstatin, Metformin, Sitagliptin, Empaglifozin, Prednisolon | Levothyroxin, Insulin, Ramipril | paroxysmal | ablation of carvotricuspid isthmus | 2016 |
| 7 | 66 | 17 | 76 | 75 | 25.1 | 25.1 | Apixaban, Bisoprolol, Pantoprazol, Atorvastatin | Apixaban, Bisoprolol | paroxysmal | PVI | 2022 |

The age is reported in years; period between MRI in months; weight in kilograms (kg); body mass index (BMI) in kg/m². Abbreviations: AF: atrial fibrillation; BMI: body mass index; ECV: electrical cardioversion; PVI: pulmonary vein isolation.
